# Point-of-View Recording Devices for Intraoperative Neurosurgical Video Capture

**DOI:** 10.3389/fsurg.2016.00057

**Published:** 2016-10-25

**Authors:** Jose L. Porras, Syed Khalid, Brandon K. Root, Imad S. Khan, Robert J. Singer

**Affiliations:** ^1^J.B. Marshall Laboratory for Neurovascular Therapeutics, Dartmouth-Hitchcock Medical Center, Lebanon, NH, USA; ^2^Dartmouth Geisel School of Medicine, Lebanon, NH, USA; ^3^Chicago Medical School, Chicago, IL, USA

**Keywords:** point-of-view, recording, streaming, Google Glass, neurosurgery, medical education, telehealth, telemedicine

## Abstract

**Introduction:**

The ability to record and stream neurosurgery is an unprecedented opportunity to further research, medical education, and quality improvement. Here, we appraise the ease of implementation of existing point-of-view devices when capturing and sharing procedures from the neurosurgical operating room and detail their potential utility in this context.

**Methods:**

Our neurosurgical team tested and critically evaluated features of the Google Glass and Panasonic HX-A500 cameras, including ergonomics, media quality, and media sharing in both the operating theater and the angiography suite.

**Results:**

Existing devices boast several features that facilitate live recording and streaming of neurosurgical procedures. Given that their primary application is not intended for the surgical environment, we identified a number of concrete, yet improvable, limitations.

**Conclusion:**

The present study suggests that neurosurgical video capture and live streaming represents an opportunity to contribute to research, education, and quality improvement. Despite this promise, shortcomings render existing devices impractical for serious consideration. We describe the features that future recording platforms should possess to improve upon existing technology.

## Introduction

The ability to record and stream neurosurgery is an unprecedented opportunity to further research, medical education, and quality improvement. Individual cases may often be performed using one of several strategies that, combined with continuous technological advances, means the operating room (OR) is in constant flux. Capturing and compiling video of these surgeries is a teaching tool that can prepare medical students, residents, and accomplished surgeons alike by providing knowledge and useful expectations of procedures, which together improve success and patient outcomes in neurosurgery. This concept is exemplified by online archives, such as The Neurosurgical Atlas, which provide high-quality video of neurosurgical procedures conducted by experienced surgeons (1).

Modern OR video recording is generally accomplished by means of specialized equipment, such as in-light cameras or modified commercially available cameras. However, existing solutions often lack utility or are impractical by nature of their design. Cameras are often unable to replicate the surgeon’s perspective during surgery and consequently, the viewer is unable to visualize the procedure in the context of complete anatomy; this is especially true of neurosurgery where the surgeon must work in relatively small spaces that obscure most recording angles. The advent of point-of-view (POV) cameras, such as the Google Glass and GoPro Hero series, now permit the recording of high-definition video captured from the surgeon’s perspective (2–4).

Google Glass is a unique POV experience because it can be operated in a hands-free manner to stream video content and communicate with remote individuals. Therefore, this device can be used to provide the surgeon’s perspective to a colleague for live consultation or education without compromising the sterile field. In 2013, the first live stream of surgery using Google Glass was performed, and this concept has since expanded to include virtually augmented surgery (5). In 2014, surgeons in the United States utilized an Apple iPad to provide on-screen annotations to a Google Glass heads-up-display during cleft lip surgery being performed in Lebanon (6).

These reports of surgical video streaming and capture using POV devices suggest that there is utility in their more widespread application into specialties, such as neurosurgery. The existence of video archives dedicated solely to the dissemination of knowledge reflects the desire and the need for neurosurgeons to expand and share their knowledge. The employment of POV cameras capable of streaming video while permitting communication is an opportunity to enrich the learning opportunities provided by previous recording technology. Despite this promise, modern POV cameras are not optimized for use in the OR. Consequently, video quality may not be optimal for capturing the level of detail required to appreciate neurosurgical anatomy. Furthermore, the device must be streamlined for use in the OR so as not to detract from the surgeon’s focus.

Our neurosurgical team performed a market analysis of available recording solutions for the OR and selected those devices representing the most promising candidates. We chose the Google Glass on the basis of its popularity and precedent in surgical streaming (7) and the Panasonic HX-A500 owing to its accessibility, resolution, and potential for custom mounting. Here, we appraise the ease of implementation of these two POV devices when capturing and sharing procedures from the neurosurgical OR, and detail their potential utility in this context. Specifically, our evaluations consider ergonomics, in-context image quality, and privacy-sensitive media sharing. Having identified existing shortcomings, we propose features that will yield improved solutions suitable for use in neurosurgery, and surgery at large.

## Methods

Our team tested the Panasonic HX-A500 during a Chiari decompression using 1,820 × 1,080/60 pixel resolution settings and during a VP shunt revision at 1,280 × 720/30 pixel resolution; both procedures were recorded under Berchtold Chromophare E558 lighting with a Luxtec headlamp also in use. We tested Google Glass during two procedures: an external carotid–internal carotid bypass performed under Berchtold Chromophare E558 lighting and a craniotomy for cavernous malformation with intraoperative angiogram performed under Berchtold Chromophare E650 BRITE halogen lighting. We critically evaluated features pertaining to ergonomics, in-context image quality, and privacy-sensitive media acquisition and sharing capacity in both the operating theater and the angiography suite. We herein refer to ergonomics as those features of a device that affect the comfort, efficiency, and safety of the neurosurgeon and patient during a procedure.

## Results

### Panasonic HX-A500

#### Ergonomics

Regarding ergonomics, the HX-A500 is compatible with loupes but is mounted laterally on the head and therefore does not align well with the surgeon’s natural gaze. The camera angle can be adjusted through 160° to mitigate this problem. However, when working in deeper tissue layers, it becomes difficult to fully appreciate the anatomy. The main unit of the HX-A500 was secured to the surgeon’s arm with the cord connecting to the camera running across the shoulder and up the neck. This was not a hindrance to mobility, but it did mean that our surgeon could not handle the device once in sterile gown. The battery captured ~144 min of footage during surgery. Given that many surgical procedures may last beyond this amount of time, a surgeon utilizing the HX-A500 must be selective with respect to what portion of a procedure to record. Given the inaccessibility of the main unit once gowned, the only means of camera control would be through the Panasonic smartphone application that enables remote control. However, micromanaging the camera’s function in this manner is undesirable as it represents a disruption to surgical workflow.

#### Media Quality

**Figure 1:**
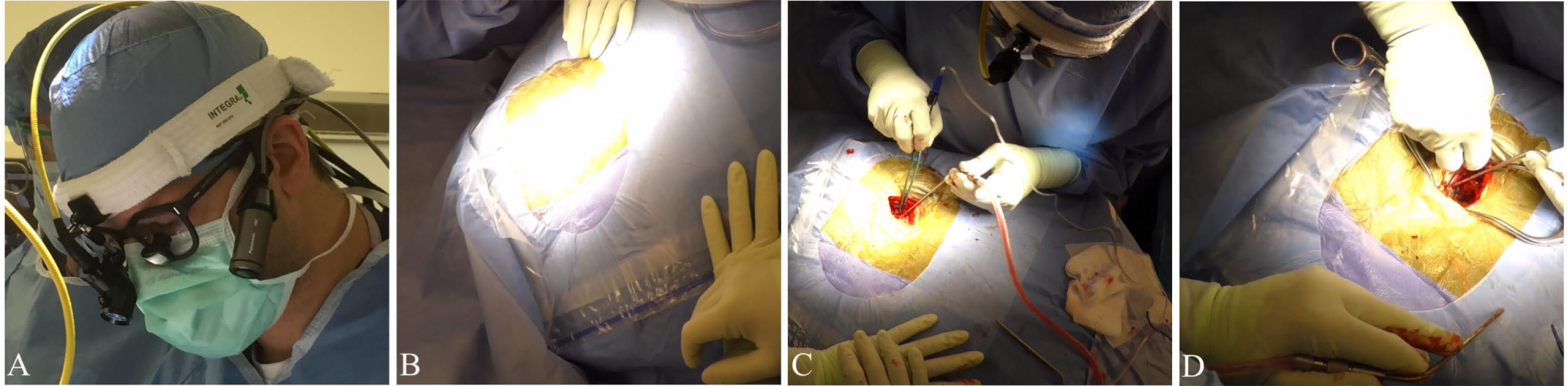
**Panasonic HX-A500**. Representative images of and video stills recorded by the Panasonic HX-A500. Video footage available in the supplement. **(A)** Image demonstrating lateral mounting of the Panasonic HX-A500. **(B)** Video still demonstrating susceptibility to halogen washout. **(C,D)** Representative stills from video clips.

The video resolution was excellent when recording with the HX-A500’s high-quality setting (1,920 × 1,080/60 pixel) and did not experience washout from halogen lighting save for short portions at the beginning and end of recordings. The device’s image stabilization aided in capturing smooth video that translated to a better appreciation for detail but was limited by a lack of zoom. Figure 1 provides representative stills captured using the Panasonic HX-A500, and a montage of representative video clips can be accessed in the references (8).

#### Media Storage and Sharing

The HX-A500 is capable of microSD/HC memory card media storage of up to 32 GB. In practice, we found this to be ~2 h and 47 min of recording high-quality video and 8 h and 40 min of recording on the lower quality setting. The HX-A500 can stream media through the use of a Panasonic application for smartphones but is not capable of voice communication. Setting up and ultimately using the HX-A500’s streaming capabilities proved cumbersome and ultimately was not tested in the OR due to institutional privacy regulations surrounding Wi-Fi use.

### Google Glass

#### Ergonomics

The Glass device excelled namely in two aspects: the fact that it is self-contained and that it can be controlled solely by the surgeon in a hands-free manner. These qualities ensure that the apparatus is not a distraction in the OR environment where the ability to focus is crucial. The heads-up-display of Glass proved to be an important complement to photography in the OR as it ensured optimal focus and positioning of the camera with minimal disruption to workflow. However, Glass was incompatible with loupes meaning that it could only be utilized for parts of the surgery. Our surgeon was able to hold the Glass device in place while wearing loupes by taping it to a surgical headlamp. This impractical solution was further complicated by the camera’s view, which is directed straight-ahead and therefore does not align with the surgeon’s natural gaze during procedures. To capture the surgical field, our surgeon was forced to look straight down so that the device was pointed in the correct direction.

By default, Glass records 10-s clips of video, and to circumvent this limitation, the user must manually swipe the device to override the default clip length. To maintain sterility, our surgeon asked an assistant to perform the manual override each time a video recording commenced. Constant recording with Glass during a procedure led to frequent overheating and a battery life of ~45 min. This was without concurrent live streaming of video content, which would likely further shorten the battery life. Like the HX-A500, the limited battery life of Glass forces the surgeon to be selective with respect to what portions of a procedure are captured if no external power source is available. The hands-free control of Glass made micromanaging recording times less of a distraction. Should the surgeon choose to circumvent the battery life limitation by maintaining power through an external source, then cables must be routed behind the surgeon, which can pose a tripping hazard.

#### Media Quality

**Figure 2:**
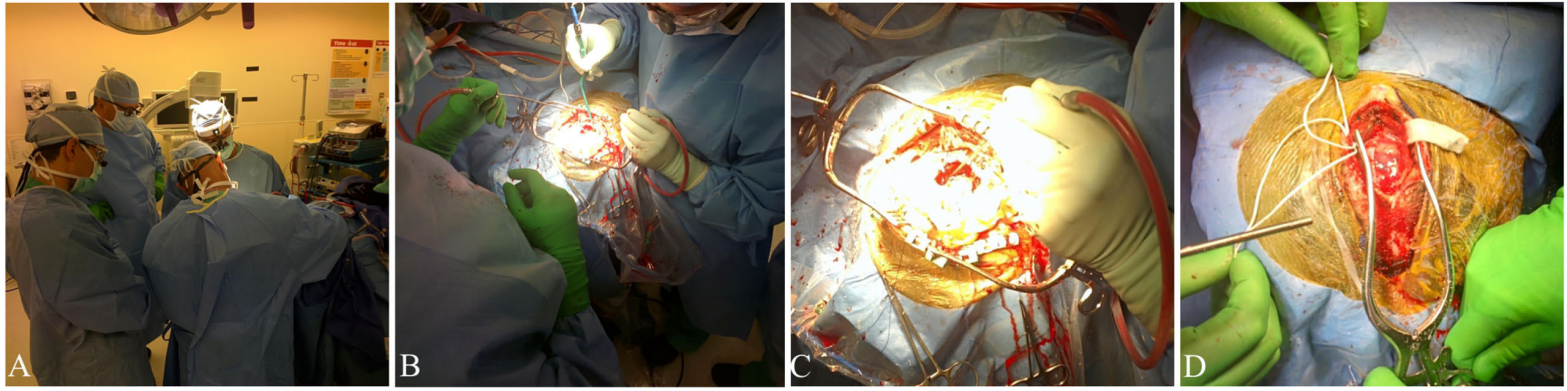
**Google Glass**. Representative images and video stills captured using Google Glass. Video footage available in the supplement. **(A)** Image of the OR. **(B)** Image captured during craniotomy for cavernous malformation. **(C)** Video still from EC–IC bypass video footage. **(D)** Image captured during EC–IC bypass.

Google Glass has a 5-MP camera, which is less than many new smartphones that feature an 8-MP camera. Footage that we captured during surgical procedures was comparable to that acquired by the average smartphone. The media also suffered from washout due to the halogen lighting. The Glass’ lack of native image stabilization also led to choppy video even when the surgeon was stationary. The Glass is also unable to zoom which, combined with other concerns over media quality renders the device suboptimal for use in procedures requiring appreciation of detailed neurosurgical anatomy. Figure 2 provides representative stills captured using Google Glass, and a montage of representative video clips can be accessed in the references (9).

#### Media Acquisition and Sharing

The Glass’ ability to capture and share media in a hands-free manner during a procedure is an important opportunity for improving communication in and out of the OR. Our surgeon had no issue using hands-free gestures to initiate capture of a photo or video clip during a procedure. As mentioned, this functionality is limited by the need for a manual override of the default 10-s clip length. The surgeon may also choose to call individuals through audio alone to provide notifications or receive a consultation. We did not stream live video from the OR with Glass due to institutional privacy regulations surrounding Wi-Fi use.

## Discussion

The present study suggests that neurosurgical video capture and live streaming through POV devices are applications through which improvements to medical education, quality improvement, and research, among other possibilities, may be improved. Despite this promise, a number of shortcomings render existing devices impractical for serious consideration. Concerns not unique to a particular device are described in the context of our evaluation categories: ergonomics, media quality, and media acquisition and sharing. Table 1 provides a qualitative summary of our Google Glass and Panasonic HX-A500 comparison. We present technical specifications of the Panasonic HX-A500, Google Glass, and a selection of other non-tested recording platforms in Table 2.

**Table 1:** **Categorical comparison of the HX-A500 and Glass**.

Parameters	Panasonic HX-A500	Google Glass
Ergonomics		✓
Loupe-compatible	Yes	No
Hands-free	No	Yes
Sterile	No	No
Remote control	Yes	Yes
Battery life	Acceptable	Poor
Media quality	✓	
Resolution	Acceptable	Poor
Fine detail	Acceptable	Poor
Media sharing and privacy		✓
Communication capable	No	Yes
Streaming capable	Yes	Yes
Wi-Fi/bluetooth	Yes	Yes
HIPAA-compliant	Yes	Yes*

*Google Glass is HIPAA-compliant following software modification to prevent communication with public Google servers.

**Table 2:** **Technical specifications of commercially available cameras for video recording and streaming in the OR**.

Parameters	Panasonic HX-A500	Google Glass	Berchtold Chromevision HD in-light camera	Panasonic AG-MDR15/MDC10	GoPro Hero series
Price	$399.99	$1,500	Not available	$3,700 + $2,500	$129.99–499.99
Resolution	3,840 × 2,160 pixel; 16 MP	720 pixel; 5 MP	1,080 pixel	1,920 × 1,080 pixel	1,080 pixel; 12 MP
Aspect ratio	16:9	16:9	16:9	16:9	16:9
Angle of view	160°	54.8°	360°	Not available	90°, 127°, or 170°
Optical zoom	No	No	10×	12×	No
Focus	Fixed	Fixed	Auto/manual	Yes	Fixed
Image stabilizer	Yes	Yes	No	Yes	No
Built-in display	No	Yes	No	Yes	Yes
Remote control	Yes	Yes	Yes	Yes	Yes
Battery life estimate	~144″	~45″	N/A	~180″	~150″
Power source	Battery, USB	Battery, USB	Light power supply	Battery, AC adapter	Battery, USB
Wi-Fi/bluetooth	No	Yes	No	No	Yes

### Ergonomics

Considering the amount of time neurosurgeons spend utilizing loupes, the inability for devices, such as Google Glass, to be worn simultaneously means that a significant portion of operations cannot be captured. The utility of existing cameras is also diminished by concerns for sterility; once gowned, a surgeon may not physically interact with a camera and in our experience, requires the aid of other OR staff for controlling basic functions. Google Glass partially mitigates this drawback by allowing the use of voice commands and head gestures, such as nods or winks, to control video recording. However, it is also reliant on the ability to swipe on the device for navigating menus. The Panasonic HX-A500 can be controlled by a remote control application used on a smart phone but does not permit head voice commands and head gestures. Battery life is a significant barrier to capturing video for significant amounts of time as it means that the surgeon is forced to be selective with respect to what portion of a surgery will be captured. When live streaming video, both the Google Glass and Panasonic HX-A500 battery life are consumed at an enhanced rate thus further constraining recording time. The fact that a surgeon must be prudent with battery life necessitates being able to control recording function, but this is made difficult by the aforementioned issue of sterility. The surgeon may opt to leave devices connected to an external power supply, but extra cords pose a tripping hazard. Furthermore, the need to micromanage any recording device is a distraction in the already hectic OR.

### Media Quality

We also found that neither the Panasonic HX-A500 nor the Google Glass capture the same level of image quality obtainable by in-light camera solutions designed for the OR. This limitation significantly impedes the utility of existing POV devices because neurosurgery requires the ability to appreciate the fine anatomical detail. These devices are also unable to capture neurosurgical procedures requiring microsurgery. The image quality of the Panasonic HX-A500 was better than that of Glass owing to its greater resolution and image stabilization capabilities, but without the ability to zoom, a surgeon cannot target specific areas of the surgical field for selective recording. Furthermore, even if the Panasonic HX-A500 was capable of zooming, the surgeon would not be able to control this function in a sterile fashion and would require remote assistance from an individual controlling the camera from a smartphone.

### Media Sharing and Privacy

The final limitation we found is that of streaming video and patient privacy. When streaming through Google Glass, the device must communicate with Google’s public servers to broadcast the signal, which is in violation of HIPAA’s standards for protecting wireless local area networks (10). Private solutions, which redirect streaming data to private servers within which patient information is secured, are available but can be cost-prohibitive and binds the user to proprietary software. The HX-A500 is not tied to public servers and can, therefore, use a hospital’s Wi-Fi to broadcast signal. However, the Panasonic camera utilized a smartphone application to achieve video streaming, which proved to be cumbersome and ultimately impractical in a neurosurgical context. Only the Glass is capable of two-way communication, which is an important feature that permits remote consultation and education. However, the streaming neurosurgeon who elects to use Glass must remain wary of additional constraints to already limited battery life.

### Discussion – Directions for Future Platforms

Despite the reported shortcomings of commercially available devices for POV recording in the neurosurgical OR, we have identified clear, achievable specifications that any future platform should possess. In the context of neurosurgery, it is especially important that cameras are optimized for maximal ergonomics, image quality, and protection of patient privacy. An ergonomic recording platform should be self-contained and operable in a hands-free manner, much like the Google Glass. There are several reasons for this, including the issue of sterility, which could be addressed through the use of an aseptic, disposable housing. The camera should require minimal time and energy investment from the surgeon. To achieve this, improved battery life and memory become critical. POV cameras used in the OR must not require a surgeon to be selective with respect to recording due to limited battery life or memory capacity – a limitation of all devices.

In this vein, future recording platforms must also be compatible with eyewear, loupes, and headlamps in such a manner that it captures the surgeon’s natural gaze without obstructing vision. The capacity for mounting the Panasonic HX-A500 to existing loupes meant that the device could be dynamically adjusted with relative ease to the surgeon’s gaze, as compared to the Glass. This is especially important given the relatively small area that a neurosurgeon must often work in – should a camera not be aligned with gaze then landmarks critical to appreciating the procedure will be missed. In addition to gaze alignment, the camera must also maintain focus on the field of view and should do so in a dynamic fashion that can account for natural movements of the surgeon’s head. We have not found dynamic gaze alignment to be a readily available feature of POV devices. The field of view should be adjustable so that either wide or narrow perspectives can be captured depending on the neurosurgeon’s needs. For the viewer, information, such as axis and angle, should be incorporated into video feed to provide orientation as the surgeon’s head moves. The relative size of structures could also be appreciated through a scale that adapts to the camera’s zoom. The wavelength response of the camera should also be optimized for function under the halogen lighting of the OR so as to avoid the media washout experienced by the Google Glass and Panasonic HX-A500.

The final specification that any successful device will feature is the ability to maintain patient privacy while allowing media streaming and communication through secure networks. The ability to receive consultation or provide viewing for educational purposes during a neurosurgical procedure is a tremendous opportunity that must remain prudent to patient privacy. Access to neurosurgical knowledge should not be restricted on the basis of geographic location, and resources, such as The Neurosurgical Atlas, demonstrate that high-quality learning materials for highly specialized fields, such as neurosurgery, can be made ubiquitous through the internet. However, bridging individuals and their knowledge and experience through the use of live video streaming can be seen as a next step in leveling access to neurosurgical consultation and medical education.

The present study is limited by the number of devices we tested. There are numerous alternative solutions available each with different technical specifications that may make them more suitable for certain OR contexts. Our study is also limited by its qualitative nature. Each camera was evaluated by our study’s own neurosurgeons. When assessing the quality of recorded media, we did not perform a blinded appraisal of the media quality and relied on our own qualitative assessment. Future studies should consider comparing more devices and blinding the device testers and those who appraise the media quality.

## Conclusion

In summary, we identify the strengths and weaknesses of a selection of commercially available POV cameras in the neurosurgical OR. We elaborate on how these aspects of existing devices can be incorporated or improved upon in future recording platforms to improve research, education, and quality in patient care.

## Author Contributions

JP – paper concept development, manuscript preparation and writing, supplementary footage editing, device testing, and manuscript submission; SK – paper concept development and manuscript preparation and writing; BR and IK – paper concept development, manuscript preparation and writing, supplementary footage editing, and device testing; RS – paper concept development, manuscript preparation and writing, supplementary footage editing, device testing, and project sponsor.

## Conflict of Interest Statement

The authors declare that the research was conducted in the absence of any commercial or financial relationships that could be construed as a potential conflict of interest.
